# Predictors of family focused practice: organisation, profession, or the role as child responsible personnel?

**DOI:** 10.1186/s12913-019-4553-8

**Published:** 2019-11-05

**Authors:** Bjørg Eva Skogøy, Terje Ogden, Bente Weimand, Torleif Ruud, Knut Sørgaard, Darryl Maybery

**Affiliations:** 1 Nordland Hospital Trust, Kløveråsveien 1, 8076 Bodø, Norway; 2 The Faculty of Health Science, UiT, The Arctic University of Norway, Box 6050, 9037 Tromsø, Norway; 3 Norwegian Center for Child Behavioral Development, Unirand, Box 7053, Majorstuen, 0368 Oslo, Norway; 4 Institute of Psychology, University of Oslo, Box 1171, Blindern, 0318 Oslo, Norway; 5 Department for Research and Development, Mental Health Services, Akershus University Hospital, Box 1000, 1478 Lørenskog, Norway; 6 Faculty of Health Sciences, Department of Nursing and Health Promotion, OsloMet – Oslo Metropolitan University, P.O. Box 4, St. Olavs plass, 0130 Oslo, Norway; 7 Institute of Clinical Medicine, University of Oslo, Box 1171, Blindern, 0318 Oslo, Norway; 8 Monash University Department of Rural Health, Box 973, Moe, Victoria 3825 Australia

**Keywords:** Family focused practice, Children as next of kin, Children of ill parents, Parental illness, Professional differences, Child responsible personnel, Policy changes, Legislation

## Abstract

**Background:**

Health professionals in Norway are required by law to help safeguard information and follow-up with children of parents with mental or physical illness, or who have substance abuse problems, to reduce their higher risk of psychosocial problems. Knowledge is lacking regarding whether organisation and/or worker-related factors can explain the differences in health professionals’ ability to support the families when patients are parents.

**Methods:**

Employing a translated, generic version of the Family Focused Mental Health Practice Questionnaire (FFPQ), this cross-sectional study examines family focused practice (FFP) differences in relation to health professionals’ background and role (*N* = 280) along with exploring predictors of parent, child, and family support.

**Results:**

While most health professions had begun to have conversations with parents on children’s needs, under one-third have had conversations with children. There were significant differences between nurses, social workers, psychologists, physicians, and others on seven of the FFP subscales, with physicians scoring lowest on five subscales and psychologists providing the least family support. Controlling for confounders, there were significant differences between child responsible personnel (CRP) and other clinicians (C), with CRP scoring significantly higher on knowledge and skills, confidence, and referrals. Predictors of FFP varied between less complex practices (talking with parents) and more complex practices (family support and referrals).

**Conclusion:**

The type of profession was a key predictor of delivering family support, suggesting that social workers have more undergraduate training to support families, followed by nurses; alternately, the results could suggest that that social workers and nurses have been more willing or able than physicians and psychologists to follow the new legal requirements. The findings highlight the importance of multidisciplinary teams and of tailoring training strategies to health professionals’ needs in order to strengthen their ability to better support children and families when a parent is ill.

## Background

Parental health and well-being are vital in the family context. Thus, physical and mental illnesses and substance abuse problems in a parent may negatively impact children in different ways: family context is a predictor of developing mental health problems [1], and genetic and environmental risks to children are associated with parental mental illness and substance abuse problems [2–4]. Evidence suggests that children whose parents have a mental illness have almost double the chance of developing a mental illness themselves [4, 5]. Parental physical illness also has a significant impact on children’s everyday lives and psychosocial adjustment [6–8], as well as an increased risk of substance abuse, mental ill-health, and criminality for one-third of children with severe parental cancer [4].

The problem also has economic implications for society. For example a register study has shown that 8% of Swedish children experienced a parent’s hospitalisation with mental illness during their childhood [4]. In adulthood, these 8% children of next of kin had developed more problems than children of parents without mental health problems and accounted for 25% of society’s annual costs of mental illness and addiction to alcohol and/or drugs [4]. If the prevalence of mental illness were the same for children as next of kin as for children in the rest of the population, societal costs would substantially decrease [4].

Since 2010, health professionals’ duties to support minors as next of kin have been enshrined in the Norwegian Health Personnel Act § 10 a [9, 10]. Health professionals treating patients with mental illness, substance abuse problems, or severe physical illness or injury are required to ‘a) register minor children in the patient’s health record, b) have conversations with the parent about children’s need for information and support, c) offer help in family information-sharing and conversations with children, d) ensure that children can visit parents at the hospital, e) assess children’s and the family’s needs, and f) gain parents’ consent to cooperate with other services in establishing necessary support’ ( [11]:2).

The Norwegian regulations have similarities to Finnish [12] and Swedish legislation [4, 13] and are in line with international recommendations to include family-focused, family-centred, family-based, family-oriented, family-inclusive, or child-centred practices to support children whose parents have mental illness [14–22], substance abuse problems [23–26], or physical illness [27–29]. The term ‘family’ may refer to the family of origin or the family of procreation [30]. This article focuses on the family of procreation: the patient/parent with an illness, the other parent /partner, and the patient’s children.

Maybery and Reupert [31] explored FFP in a literature review and developed a measure to study barriers and enablers of the mental health workforce’s ability in FFP [32, 33]. The theoretical perspective [33] has been informed by the literature on family-centred practice, with the origin based on Dunst [34]. FFP describes a continuum of practices to support the whole family unit, both the parents and the children [14, 20].

In this study, we explore FFPs as required by the new legislation [9, 10], regarding children as next of kin, which in 2017 was supported by research-based national guidelines on next of kin (children and adults) for Health and Care Services [35].

### Positive effects of family focused interventions

There is increasing evidence of the positive effects of family focused interventions when parents are ill. A meta-analysis of 13 individual, group, and family interventions for families with parental mental illness has found a reduced risk of children developing the same illness as their parents by 40% [36]. These interventions have been found to increase parenting skills, strengthen knowledge of parents’ mental disorders, and strengthen resilience factors among adolescents [36].

Systematic reviews of prevention programs for the children of parents with substance abuse problems [26, 27] have found preliminary evidence on reducing children’s problems and improvements in positive behaviours, coping skills, and feelings, especially in longer programs that involved both parents and children [26].

Similarly, a systematic review of 19 psychosocial interventions for families with parental cancer found most interventions helpful [37]. The interventions were found to support more open communication in the families and children reported to talk more openly about parental illness and have better coping strategies [37]. Studies also showed improvements in parents’ and children’s quality of life, mental health or distress [37].

### Change agents to promote FFP

Change agents [38] or champions [39] have been found to play an important role in innovative practice, and an internal organisational champion increases the likelihood that a new practice is implemented [40, 41]. Rogers ( [39]:992) has defined a champion as ‘an individual who devotes his/her personal influence to encourage adoption of an innovation’. Fixsen et.al. ( [42]:14) have defined purveyors, also called change agents or implementation teams [43], as ‘an individual or group of individuals representing a program or practice who actively work to implement that practice or program with fidelity and good effect’. To implement a new policy, policy makers often try to identify champions who could become local change agents within the organisations [44].

As part of the changes in legislation in Norway, Specialised Health Services are obligated to have CRP to promote and coordinate health professionals’ support of parents, in their parental role, and of their children [9]. Across Norwegian hospitals, we found that 1429 health professionals were appointed to the CRP role (as part of their ordinary position), usually one or two per unit [11, 45]. To coordinate training and supervision, hospital coordinators (H-CRP) have been established, usually in a 20–50% role as part of another position [46].

Other countries have also used change agents to implement changes in legislation and encourage FFP. In Finland, a ‘train the trainers model’ was developed to make clinicians ‘early adopters’ [39] and interest personnel in expanding the work [47]. Like Norway, in 2010, Sweden appointed children’s representatives/spokespersons (Barnrättsombud/barnombud) in hospitals to support the development and implementation of legal changes [48]. In Victoria, Australia, the Families Affected by Mental Illness (FaPMI) strategy has created FaPMI coordinators across 11 regions to organise training and networking that encourage FFP [21].

### Differences in FFP between professions

Despite the need for family focused interventions, it has been shown that different professions provide different levels of FFP [49, 50]. Differences in learning needs have been found [51], and mental health nurses, psychologists, and physicians want more knowledge on how mental illness can affect the parenting role and on how to support families. Australian social workers in adult mental health services have been found to be more confident in working with families, parents, and children and provide FFP than psychiatric nurses [49]. Additionally, both social workers and psychologists provided more direct family support (e.g., psychoeducation and family meetings) and referrals than psychiatric nurses did [49]. In Thailand, social workers in mental health services provided more FFP than psychiatric nurses, psychologists, and psychiatrists did, with social workers referring more to other services than nurses and psychologists [50]. Participants who had received previous family and child-focused training scored highest [50].

In Germany, many physicians treating parents with cancer were reluctant to refer families to Children of Ill Parents (COSIP) therapists [52], and physicians concerns about their own resources and patients’ well-being were common problems in the implementation of preventive mental health services for children of physically ill parents in European countries [29]. In Norway, general practitioners who were positive to support to children, often forgot to address the children’s needs or were afraid of increasing the parent’s feelings of guilt and shame [53].

More generally, the organisational culture and climate has been associated with clinicians’ attitudes to adopt evidence-based practices (EBP) [54]. Aarons et al. [54] have found four dimensions in clinicians’ attitudes towards EBP: the intuitive appeal, the perceived difference between the current and the new practice, the likelihood of following new institutional requirements, and general openness to learning new practices.

### Facilitators and predictors of FFP

Both organisation-related and worker-related factors have been associated with health services’ possibilities of providing family support. Organisation-related factors such as establishing mentoring and supervision have been found to be important enablers of family focused practice [31, 55, 56], and co-worker support and time predict family support [55]. Location (e.g., a rural area) has been identified as a predictor of family support, with services available predicting referrals [55]. A number of worker-related predictors of family and parenting support have been identified; knowledge and skills, further training, and connectedness predict family support, while skills and knowledge, connectedness, and engagement with family members predict referrals [55].

Well-trained and rurally located practitioners have been found to predict FFP [57], as well as knowledge and skills, followed by confidence [58]. Practitioners’ gender, age, and length of experience have also been found to have an impact, but the results differ. Some studies have found that younger health professionals with medium education have more positive attitudes toward supporting mentally ill parents in their parental role and supporting their children [59], and more inexperienced psychiatric nurses were more likely to support service users’ children [60]. Other research has found that female, older, married, and experienced mental health nurses engaged themselves more in FFP [61], and their own parenting experiences were a key predictor [58].

Effective collaboration with clinics and institutions, location, intervention characteristics, and provision of information about support services can facilitate families’ use of psychosocial support services [37]. Support as part of routine care, as well as having a contact person in clinics, could facilitate the use of support for patients and families [29, 52, 62].

While numerous barriers to FFP and the use of psychosocial support have been identified in previous research [20, 31, 37, 55, 56, 59, 61], less is known about factors that facilitate and predict health professionals’ ability to engage in FFP, particularly in relation to new policies and guidelines, such as those recently introduced in Norway.

A few studies have identified differences in professional background related to performing FFP [49, 50]. Some smaller studies have also discussed the new role of the CRP within mental health services [63–65]. Studies of parental cancer, meanwhile, have identified facilitators of using psychosocial support [37], but earlier studies of predictors of FFP have been limited to mental health services. Some studies have explored predictors of FFP [55, 57–59]; however, these have not included professional background as a predictor of FFP, and they are also limited in their explanations of lower levels of variance [55].

To date, no research has studied the relative importance of organisation-related factors and worker-related factors of FFP, in addition to demographics, professional background, and the role of CRP as predictors of FFP. The present study is also the first to include health professionals from mental health, physical health, and substance abuse settings regarding FFP. Such information is important to be able to develop training programs and tailor improvement strategies to health professionals’ needs.

## Aims

The first aim was to analyse and compare differences of FFP in personnel with different professional backgrounds; second, this study analyses and compares differences of FFP between health professionals appointed to the role of CRP and other clinicians. Finally, the third aim was to explore predictors of FFP among health professionals.

## Method

### Design

This study was part of an exploratory, cross-sectional, multi-centre study, the Children of Ill Parents (CHIP)-study [45], with data collected from patients that were parents, the other parent/adult [66–69], children and adolescents (8-18 years) [69–72], children’s teachers, health professionals treating the ill parents, health professionals in the role of CRP, managers/ leaders, and hospital coordinators (H-CRP) [11, 46].

In the current study, only data from health professionals about their FFPs are reported.

### Context

The five hospitals in this study serve 34% of the total Norwegian population of 5.2 million. Hospital 1 (H1) serves 136,000 inhabitants, H2 serves 290,000, H3 serves 358,000, H4 serves 480,000, and H5 serves 493,000 [73]. Hospital 1 is the smallest hospital and provides health services to a large rural area, while Hospitals 3 and 5 are university hospitals.

### Sample

The 280 health professionals who participated in the current study were recruited from stratified, randomly selected inpatient and outpatient units at the five hospitals from mental health, physical health (cancer and neurology), and substance abuse units for adult patients. The first group was health professionals who were CRP (*n* = 104, 72% response rate), one per unit. The second group consisted of other clinicians (C), (*n* = 176, 52% response rate) treating patients who were recruited for the larger part of the CHIP study. Among these, 32 were also CRP, who were added to the CRP above, resulting in two groups of health professionals, CRP *(n* = 136) and C (*n* = 144).

The participants were mostly female with significant experience, with more nurses and psychologists participating than social workers, physicians, and others (Table 1). Other participants included family therapists, physiotherapists, occupational therapists, hospital chaplains, and nurse assistants.

**Table 1:** Descriptive statistics of Demographics and Professional background on Child Responsible Personnel (CRP) and Other Clinicians (C), *N* = 280

	Total	CRP (*n* = 136)	C (*n* = 144)	*p*
Age (SD)	45.4 (10.2)	45.53 (9.90)	45.31 (10.45)	.854
Yrs experience (SD)	18 (10.1)	19 (10.04)	17 (10.17)	.243
Yrs in post (SD)	6.1 (5.6)	6.61 (5.98)	5.61 (5.25)	.138
Gender				
Women (%)	224 (80)	122 (90)	102 (71)	.001*
Men (%)	56 (20)	14 (10)	42 (29)	.001*
Profession				
Nurse (%)	101 (36.1)	64 (47.1)	37 (25.7)	.001*
Psychologist (%)	71 (25.4)	17 (12.5)	54 (37.5)	.001*
Social worker (%)	42 (15.0)	31 (22.8)	11 (7.6)	.001*
Physician (%)	32 (11.4)	1 (0.7)	31 (21.5)	.001*
Other (%)	34 (12.1)	23 (16.9)	11 (7.6)	.018*
Specific training				.000*
No (%)	95 (34.2)	32 (23.5)	63 (43.8)	CRP < C
Some degree (%)	80 (28.6)	33 (25.0)	46 (31.9)	
Yes (%)	105 (37.8)	70 (51.5)	35 (24.5)	CRP > C

Note, *p < .05

There were significant differences between CRP and other clinicians (C) based on gender, professional background, and specific training. Of the CRP, there were more women and fewer physicians and psychologists, and the CRP had received more specific training after the changes in legislation.

### Data collection

The data was collected from June 2013 to December 2014; health professionals received an e-mail invitation with reminders. A link and password to the electronic questionnaire were distributed after informed confirmation of participation, and the participants gave a written consent on the first page of the survey.

### Measure

The measure employed in this study was adapted from the Family Focused Mental Health Questionnaire [33] and is based on a literature review [31, 32]*.* The questionnaire has been employed in Australia, specifically in regard to FFP in relation to parental mental health problems [49, 74, 75], and in Ireland [56], Thailand [50], and Norway [11, 59, 76]. The 49-item measure with 17 subscales [33] employs a seven-point Likert Scale. Scores ranged from 1 to 7, from *strongly disagree* = 1 to *strongly agree* = 7, in addition to *not applicable* (N/A).

The measure was translated into Norwegian following the steps of back-translations [77] and made generic in order to focus on health professionals’ work with parents affected by all kinds of illnesses [11].

In the introduction to the generic version of the questionnaire it was stated that the aim was to explore *family focused practice* as required by the new Norwegian legislation, and included *all types of illnesses* (mental, physical, and substance abuse), and the questionnaire was slightly reworded (e.g. mental illness was replaced by illness) [11]. The questionnaire was tested for content validity in a sample of experts in this area, and a pilot study was conducted to test the clarity of the questions and the layout in a group of health professionals and user consultants [11].

The reliability of the measure was analyzed using Cronbach’s alpha with SPSS (version 24). Scale Reliability Analysis suggested the deletion of three items before the factor analysis, which gave higher reliability on three of the scales (need training, confidence, and family support). The reliability of the subscales ranged from .17 to .80, with seven scales scoring below .60 [11], which confirmed low scores on three subscales the developers also found [33]. In this article, we report only the 10 subscales (31 items) that scored over .60 [78, 79]: workplace support: .67, co-worker support: .62, service available: .62, family support: .67, referrals: .69, and five that scored over .70: connectedness: .71, confidence: .72, need training: .74, knowledge and skills: .76, time and workload: .80.

Health professionals were also asked three additional questions: 1) how many conversations they had with parents, 2) how many conversations they had with children, and 3) how many parents had refused conversations with their children in the last 2 months. These were rated as follows: *none = 0, one to two = 1, three to five = 2, over five = 3.* Health professionals were also asked if they had participated in specific training to deliver FFP according to the changes in the law. These where rated *no = 0, to some degree = 1, yes = 2.*

### Analysis

The electronic questionnaire did not allow any missing values. Most subscales had less than 5% for N/A, except for *family support* and *referrals*, indicating that family support and referrals were not appropriate or necessary for some families. The N/As were not included in the regression analysis.

Descriptive statistics and chi-square tests were used to calculate differences of demographics and professional background between CRP and other clinicians (Table 1).

A one-way between-groups analysis of variance (ANOVA) was conducted to determine the impact of professional background on FFP, as measured on the FFPQ (Table 2). Descriptive statistics and chi-square tests were used to determine the impact of professional background on health professionals having conversations with parents, children, and parents refusing conversations (Table 3). Next, a two-way between groups analysis of covariance (ANCOVA) was performed to determine health professionals’ role (CRP and C) on the level of FFP, controlling for the demographics, professional background, and having received specific training. Professional backgrounds were coded as dummy variables, with ‘nurse’ as the reference category. There was no statistically significant interaction effect between the role of personnel and hospitals on any of the subscales; therefore, differences between CRP and C are reported here (Table 4).

**Table 2:** Mean differences (ANOVA) of Family Focused Practice Subscales on Professional Background (*N* = 280)

	Total	1	2	3	4	5		
		Nurse	Psych.	Physician	SocWkr	Other		
		*(n = 101)*	*(n = 71)*	*(n = 32)*	*(n = 42)*	*(n = 34)*		
	*M(SD)*	*M(SD)*	*M(SD)*	*M(SD)*	*M(SD)*	*M(SD)*	*p*	*Profession*
Organisation								
Workplace support	4.51 (1.54)	4.66 (1.56)	4.48 (1.44)	4.41 (1.30)	4.07 (1.86)	4.72 (1.55)	.349	
Co-worker support	5.05 (1.13)	5.27 (1.03)	5.09 (.94)	4.68 (1.19)	4.86 (1.40)	4.91 (1.26)	.064	
Time family work	4.48 (1.45)	4.93 (1.34)	3.96 (1.36)	3.76 (1.35)	4.86 (1.37)	4.54 (1.55)	.000*	1,4 > 2,3
Service available	4.84 (1.34)	4.89 (1.32)	4.87 (1.30)	3.91 (1.23)	5.29 (1.29)	5.00 (1.35)	.000*	1,2,4,5 > 3
Worker								
Knowledge skills	4.90 (.99)	5.00 (.96)	4.87 (.87)	4.19 (1.23)	5.19 (.72)	5.02 (1.09)	.000*	1,2,4,5 > 3
Connectedness	5.12 (.95)	5.13 (.94)	5.28 (.88)	4.58 (1.10)	5.23 (.81)	5.13 (1.03)	.012*	1,2,4 > 3
Confidence	5.68 (1.15)	5.88 (1.02)	5.36 (1.22)	5.24 (1.23)	5.96 (1.14)	5.84 (1.06)	.003*	1 > 2
Need training	5.42 (1.05)	5.45 (1.16)	5.41 (1.01)	4.97 (1.01)	5.44 (.97)	5.44 (.97)	.073	
Practice								
Family support	3.85 (1.24)	4.09 (1.17)	3.03 (1.05)	3,43 (1.37)	4.65 (1.18)	3.68 (1.07)	.000*	4 > 2,3,5 1 > 2
Referrals	4.08 (1.57)	4.31 (1.51)	3.58 (1.38)	3.09 (1.47)	5.12 (1.31)	4.31 (1.61)	.000*	1,4,5 > 3 1,4 > 2

Note, * *p* < .05, FFPQ, range 1–7,

**Table 3:** Conversations with Parents, Children and Parents Refusing Conversations, Last Two Months (*N* = 280)

	Nurse	Psych.	Physician	SocWkr	Other	Total	*χ* ^*2*^	*df*	*p*
Conv. Parents							21.059	12	.050
0	21 (20.8)	5 (7.0)	4 (12.5)	8 (19.0)	6 (17.6)	44 (15.7)			
1–2	32 (31.7)	20 (28.2)	13 (40.6)	8 (19.0)	15 (44.1)	88 (31.4)			
3–4	24 (23.8)	31 (43.7)	10 (31.3)	13 (31.0)	5 (14.7)	83 (29.6)			
> 5	24 (23.8)	15 (21.1)	5 (15.6)	13 (31.0)	8 (23.5)	65 (23.2)			
Total	101 (100)	71 (100)	32 (100)	42 (100)	34 (100)	280 (100)			
Conv. Children							9.953	12	.620
0	70 (69.3)	56 (78.9)	23 (71.9)	28 (66.7)	25 (73.5)	202 (72.1)			
1–2	24 (23.8)	10 (14.1)	8 (25.0)	9 (21.4)	7 (20.6)	58 (20.7)			
3–4	7 (6.9)	4 (5.6)	1 (3.1)	3 (7.1)	1 (2.9)	16 (5.7)			
> 5	0 (0.0)	1 (1.4)	0 (0.0)	2 (4.8)	1 (2.9)	4 (1.4)			
Total	101 (100)	71 (100)	32 (100)	42 (100)	34 (100)	280 (100)			
Parents refused							9.088	12	.695
0	68 (67.3)	41 (57.7)	24 (75.0)	27 (64.3)	25 (73.5)	185 (66.1)			
1–2	27 (26.7)	23 (32.4)	7 (21.9)	9 (21.4)	6 (17.6)	72 (25.7)			
3–4	4 (4.0)	6 (8.5)	1 (3.1)	4 (9.5)	2 (5.9)	17 (6.1)			
> 5	2 (2.0)	1 (1.4)	0 (0.0)	2 (4.8)	1 (2.9)	6 (2.1)			
Total	101 (100)	71 (100)	32 (100)	42 (100)	34 (100)	280 (100)			

Note, χ^2^_= chi-square,_

**Table 4:** Differences in Family Focused Practice between C and CRP (ANCOVA), Controlling for Demographics, Professional Background, Hospitals, and Receiving Specific Training (*N* = 280)

	C (*n* = 144)	CRP (*n =* 136*)*					
	*M* (95% CI)	*M* (95% CI)	*Mean difference*	*df*	*t*	*eff. Size*	*p*
Organisation							
Workplace support	4.68 (4.38–4.98)	4.31 (4.02–4.60)	−.370	1.235	2.604		.108
Co-worker support	4.96 (4.75–5.18)	5.09 (4.88–5.31)	.126	1.249	.571		.451
Time family work	4.27 (4.00–4.53)	4.58 (4.31–4.84)	.310	1.246	2.296		.131
Service available	4.80 (4.56–5.05)	4.94 (4.69–5.15)	.131	1.248	.478		.490
Worker							
Knowledge skills	4.75 (4.58–4.92)	5.09 (4.92–5.26)	.339	1.249	6.615	.03	.011*
Connectedness	4.98 (4.81–5.16)	5.21 (5.03–5.39)	.228	1.250	2.855		.092
Confidence	5.45 (5.24–5.66)	5.91 (5.70–6.12)	.458	1.249	7.753	.03	.006*
Need training	5.41 (5.22–5.61)	5.41 (5.22–5.60)	−.006	1.243	.001		.970
Practice							
Family support	3.77 (3.55–4.00)	3.85 (3.62–4.07)	.073	1.218	.179		.672
Referrals	3.87 (3.58–4.15)	4.34 (4.04–4.64)	.475	1.208	4.395	.02	.037*
Conv. Parents	1.54 (1.48–1.85)	1.66 (1.48–1.85)	.123	1.258	.753		.386
Conv. Children	.30 (.18–.43)	.40 (.28–.53)	.101	1.258	1.099		.295
Parents Refusals	.40 (.28–.53)	.51 (.38–.64)	.105	1.258	1.129		.289

Note, *M* = Adjusted means, controlling for covariates, * *p* < .05, FFPQ, range 1–7, Conversations with parents, children, and refusals, none = 0, one to two =1, three to five = 2, over five = 3

Finally, correlations between 16 organisation-related and worker-related factors, used as independent variables (IV), and five practice-related behaviours, used as dependent variables (DV), were explored, followed by a multiple regression analysis (Table 5). The length of experience was included as IV, but not age, because these were highly correlated (*r* = .88). As there should be 10–15 cases per predictor [80], using a parameter of 16 independent variables and 280 cases should have been suitable for the regression analysis.

**Table 5:** Predictors of Family Focused Practice Behaviours, Summary Table (*N* = 280)

DV	Significant predictors	*B*	*SE B*	*β*	*Adj.R* ^*2*^	*df*	*F*	*p*
Conversations with parents	(Constant)	32.84	13.61		.211	16.216	4.861	.001
	Knowledge Skills	.31	.09	.31				.001***
	Experience (low)	−.02	.01	−.18				.012*
	Gender (female)	.42	.15	.18				.006**
Conversations with children	(Constant)	7.18	9.95		.109	16.216	2.762	.000
	Co-Worker Support	.09	.05	.16				.039*
Parents refusing conv. Children	(Constant)	−21.62	10.70		.095	16.216	2.566	.001
	Knowledge Skills	.23	.07	.32				.002**
	Gender (female)	.28	.12	.16				.018*
Family support	(Constant)	−11.03	20.60		.454	16.201	12.294	.000
	Time Family Work	.18	.05	.21				.001***
	Specific Training	.29	.09	.19				.001***
	Social worker	.65	.20	.19				.002**
	Psychologist (not)	−50	.20	−.18				.012*
	Co-worker Support	.16	.07	.14				.021*
	Connectedness	.18	.09	.14				.045*
	Gender	.38	.16	.13				.021*
Referrals	(Constant)	9.53	15.24				9.122	.000
	Service Available	.33	.07	.29				.000***
	Knowledge Skills	.29	.14	.19				.041*
	Social worker	.60	.28	.14	.387	16.186		.034*

Note, *DV* =  Dependent variables. Professions were represented as four dummy variables, with ‘nurse’ serving as the reference group. Only significant predictors are shown. *** *p* < .001, ** *p* < .01, * *p* < .05

## Results

### Differences of FFP on professional background

The ANOVA showed significant differences between the type of professional background on seven FFP subscales; see Table 2. Physicians scored the lowest on five subscales: time for family work, services available, skills and knowledge, connectedness, and referrals; however, they had moderate scores on family support. Social workers scored significantly higher on family support than psychologists, physicians, and others, with nurses scoring higher on family support than psychologists. Both social workers and nurses scored significantly higher on referrals than psychologists and physicians.

### Differences in conversations with patients as parents, their children, and parents refusing conversations with their children

Descriptive statistics showed that most health professionals had begun having conversations with parents about children’s needs, but only one-third have had conversations with children. There were differences between the types of professions regarding how many conversations they had with parents, but these were slightly not significant.

### Differences in FFP between CRP and other clinicians

In the ANCOVA, when controlling for demographics, there was a significant difference between CRP and other clinicians on three subscales: knowledge and skills, confidence, and referrals; see Table 4. The effect sizes were in a medium range (0.10 = small, 0.25 = medium, 0.40 = large) [81]. Other significant effects for *skills and knowledg*e included age, specific training, and type of hospital. There were no other significant effects for *confidence*. Other significant effects for *referrals* were specific training and professional background, with social workers providing significantly more referrals than nurses.

### Predictors of FFP behaviours

The five family focused practice behaviours (family support, referrals, conversations with parents, conversations with children, and parents refusing conversations with children) were used as dependent variables, and 16 organisation-related and worker-related factors served as independent variables.

#### Correlations between variables

There were significant, positive associations between family support and referrals (*r* = .59), conversations with parents and conversations with children (*r* = .42), and conversations with children and family support (*r* = .36). In addition, parents refusing conversations with their children was significantly associated with conversations with parents (*r* = .38), family support (*r* = .36), and knowledge and skills (*r* = .23). The five FFP behaviours were significantly and positively associated with many of the organisation-related and worker-related variables, with the highest associations to knowledge and skills, connectedness, confidence, and gender (female).

#### Regression analyses

Regression analyses were conducted by employing five dependent variables; they are shown in Table 5. Only the significant predictors are shown. Additional tables with all predictors included are available in the Additional file 1: Table S1a-e.

Building on Maybery et al.’s model of organisation-related factors as a basis of family support [31, 55], these factors (workplace support, co-worker support, time for family work, and service available) were included first (model 1) before adding the worker-related factors: profession (nurse, social worker, psychologist, physician, and others, model 2), role (CRP or C, model 3), demographics (gender and length of experience, model 4), and other worker-related factors (knowledge and skills, connectedness, confidence, and need training, model 5).

In regard to health professionals having conversations with parents about children’s needs, the multiple linear regression analysis indicated an equation of *F*(16.216) = 4.861, *p* < .001, with *R*^*2*^ of .270 and adjusted *R*^*2*^ of .211 for the largest model. In this model, 21% of the variance was explained, with 5% explained by organisation-related factors, and the additional 16% explained by the worker-related factors. The significant predictors in order of weight (beta values) were knowledge and skills, length of experience (lower), and gender (female).

For health professionals having conversations with children, the multiple linear regression analysis indicated an equation of *F*(16.216) = 2.762, *p* < .001, with *R*^*2*^ of .170 and adjusted *R*^*2*^ of .109 for the largest model. In this model, 11% of the variance was explained, with 6% explained by organisation-related factors, and the additional 5% explained by worker-related factors.

Concerning parents refusing conversations with children, the multiple linear regression analysis indicated an equation of *F*(16.216) = 2.566, *p* < .001, with *R*^*2*^ of .158 and adjusted *R*^*2*^ of .095 for the total model. In this model, 10% of the variance was explained, with 2% explained by organisation-related factors, and the additional 8% explained by worker-related factors. The significant predictors in order of weight (beta values) were knowledge and skills, and gender (female).

The multiple linear regression analysis of health professionals delivering family support indicated an equation of *F*(16.201) = 12.294, *p* < .001, with *R*^*2*^ of .495 and adjusted *R*^*2*^ of .454 for the total model. In this model, 45% of the variance was explained, with 33% explained by organisation-related factors and the additional 12% explained by the worker-related factors. The significant predictors in order of weight (beta values) were time for family work, specific training, being a social worker, not being a psychologist, co-worker support, connectedness, and gender (female).

For health professionals making referrals for children or families, the multiple linear regression analysis indicated an equation of *F*(16.186) = 9.122, *p* < .001, with *R*^*2*^ of .435 and adjusted *R*^*2*^ of .387 for the total model. In this model, 39% of the variance was explained, with 31% explained by the organisation-related factors, and the additional 8% explained by worker-related factors. The significant predictors in order of weight (beta values) were service available, knowledge and skills, and being a social worker.

In summary, worker-related factors such as knowledge and skills and gender (female) were key predictors of having conversations with parents and parents refusing conversations with children, with knowledge and skills also predicting referrals. Specific training and gender (female) predicted family support. Profession was also a key predictor of family support and referrals, but the role of being CRP did not predict either family support or referrals. The only predictor of having conversations with children was the organisation-related factor co-worker support. Both organisation-related factors and worker-related factors were key predictors of family support and referrals, with more of the variance explained by organisation-related factors than by worker-related factors.

## Discussion

This is the first study to investigate FFP of health professionals in all types of services (mental health, physical health, and substance abuse). There were significant differences between nurses, social workers, psychologists, and physicians on seven of the FFP subscales, with overall better scores from social workers and nurses.

In addition, when health professionals appointed to the role as CRP were compared to other clinicians (controlling for demographics, professional background, and specific training), CRP scored significantly higher on three subscales: knowledge and skills, confidence, and referrals. Significant predictors of FFP varied between the less complex practices (talking with parents) and the more complex (family support and referrals).

### Differences of FFP in professional background

One reason for the higher number of conversations with parents about children’s needs than having child and family conversations may be that health professionals (especially the physicians and psychologists) perceive this practice as less different from their current practice, and it may have more intuitive appeal, both of which are important aspects of health professionals having positive attitudes to delivering new EBPs [54].

Compared to other countries, Norwegian nurses (in all types of services) scored higher overall on FFP and delivered more family support and referrals than mental health nurses in Ireland [56], Thailand [50], and Australia [49]. While social workers in other countries were found to score higher on FFP than other professions [49, 50], an interesting finding is that both social workers and nurses in Norway gave more family support than psychologists, and they also made more referrals than both psychologists and physicians. Physicians scored the lowest on many of the FFP subscales but had moderate scores on family support. It is possible that nurses in Norway have more family focus in their undergraduate training than in other countries, or that they have responded more rapidly to the requirements of the changes in the law compared to health professionals with a longer education.

Another possibility is that physicians and psychologists may rely more on their autonomous decision-making or may be more reluctant to meet new requirements, which is in line with implementation studies of evidence-based practices, in which practitioners with higher levels of education scored lower on new requirements [54, 82, 83].

Alternatively, physicians and psychologists may consider that they have less time to support families or may not consider it an important component of their role with patients. As professions, physicians and psychologists in the hospitals may be more likely to have their primary focus on ‘the identified patient’, so that the expectation to consult with children would be a significant deviation from their usual practice and role. By contrast social workers may be more likely to see ‘the patient as part of the family context’, with the new legislative expectations being more like their usual practice. This may be an important focus for future research.

### Differences of FFP on health professionals’ roles

Trying to change the entire workforce to deliver more FFP is difficult, and one particular approach to implementing the new legislation in Norway was to require hospitals to appoint CRP to promote and coordinate support given by health professionals to parents in their parental role and to their children. As expected, according to their role, CRP had received significantly more specific training than the other clinicians. Notably, CRP included more nurses and social workers.

This study showed significant differences between the two groups (C and CRP) on the FFP subscales knowledge and skills, confidence, and referrals, controlling for demographics, type of hospital, and whether they had received specific training about health professionals’ new legislative duties. The findings indicate that CRP are selected for this role, and that highly skilled and motivated champions may have volunteered or been appointed by their leaders to take on the role of change agent. In accordance with their new role, this suggests that the CRP are in a position to supervise and support other health professionals in family and child conversations, and they have the potential to spread FFP within the hospitals.

Qualitative studies from mental health services in Norway have found that CRP develop their role differently; some are ‘watchdogs’ for colleagues trying to promote parents’ and children’s needs [64, 84], while others develop a ‘family expert’ role, taking on most family work themselves, or they can experience their work as a lonely, ‘unclear role’ [64, 85].

The current study (4 years after the policy changes) contrasts with some of the earlier findings, and do not indicate that CRP take most family work themselves. This supports other findings from the CHIP study, in which 80% of health professionals said that CRP kept them updated about legislation and guidelines, and 52% said that CRP supported them in family conversations [45]. CRP scored significantly higher than other health professionals on referring children and families for further support, which is in line with 86% of CRP stating that they are highly knowledgeable about other services available [45]. This suggests that many CRP have developed an ‘information and supervision’ role, and they are in a position to motivate and support other health professionals, in addition to taking action when children and families need further support.

However, one weakness is that fewer psychologists and physicians have this type of role. Change agents within their own profession may have more potential to strengthen the spread of FFP among their peers [63].

Another part of CRP’s role is to systematise hospitals’ follow-up regarding the new law and guidelines. As most hospitals have hospital coordinators (H-CRP) and one or two CRP in each unit [45, 46], CRP are in a unique position to systematise the work in their unit together with their leaders. Leadership and resources to establish inner support and to collaborate with outer systems have been found to be key predictors of implementation satisfaction [46], and H-CRP play an important role in systematising the hospitals’ work and collaboration with external systems [46].

### Predictors of FFP behaviours

Significant predictors of FFP varied between the less complex (talking with parents) and the more complex practices (family support and referrals).

The most important predictor for having conversations with parents regarding their children’s needs was perceived skill and knowledge. Having received specific training and being able to determine connectedness (parental awareness of child connectedness) were key predictors of family support. This highlights the importance of the knowledge and skills needed to be able to support parents and families, which is consistent with previous research [55, 57, 58].

The second predictor of conversations with parents was length of experience. This finding suggests that newly trained (and younger) health professionals are more open to the changes in law, which supports other research showing that younger health professionals have more positive attitudes to supporting mentally ill parents and their children [59].

However, the more complex behaviour of delivering family support is not associated with being newly trained or with younger health professionals. Another notable finding is that women had more conversations with parents and delivered more family support. This partly supports earlier studies, in which female, older, married, and experienced mental health nurses engaged themselves more in FFP [61].

Co-worker support was the only significant predictor of having conversations with children, and it was also a significant predictor of family support, which aligns with earlier research [55, 58]. This indicates that conversations with children and families are better achieved if health professionals have the opportunity for supervision and work in multidisciplinary teams. The implementation literature [42, 86] and studies on FFP confirm this finding [31, 47, 87, 88]. To establish supervision, leadership support and resources are needed [46]. Other organisation-related predictors of family support and referrals were that the health professionals had time for family work and that services were available to refer children and families to for further support, which also aligns with existing research [55].

In sum, these findings highlight that family support, such as psychoeducation and having family meetings, is more complex than having conversations with parents regarding children’s needs. To some degree, this is a unique finding that highlights that not all FFPs are the same. To increase family support and referrals for additional support, it is important to address a number of organisation-related and worker-related factors. Future research could expand this study to examine the requirements for different types of FFPs.

### Strengths and limitations

A key strength of the study is that the two groups of personnel were recruited from stratified, randomly selected inpatient and outpatient units, from mental health, substance abuse, and physical health in five hospitals that covered 34% of the population, which suggests that the findings could be generalised across hospitals in Norway [11, 46].

A potential weakness was that the response rate for CRP was high (73%), but lower (52%) for the second sample of clinicians who were responsible for treating patients recruited for the larger part of the CHIP study [11]. Health professionals in the second group may have been less interested to contribute on this topic, or the lower participation, especially from the psychologist and physicians, may be caused by higher workload [11]. Moreover, the FFP data relied on personnel self-reports, which may not reflect actual practice. Another limitation was the unequal sample size across groups of health professions (*n* = 32–101), which may decrease the probability of finding statistically significant evidence in the smaller groups [89].

A strength is that the Family-Focused Mental Health Questionnaire has been used in other countries, which creates the ability to compare outcome differences, although some caution should be exercised in comparing concepts across countries [90]. Only 10 of the 17 subscales had high enough reliability to be further analysed in this study [78], which contributes to knowledge on the weaknesses of some subscales [33, 56, 75, 91] and provides useful information on refining the measure.

As this is a cross-sectional study, it is important to notice that the relationship found in the regression analysis may not be causal. Another important weakness is the lower levels of variance explained by the regression equation for conversations with parents (21%), conversations with children (11%), and refusals of conversations with children (10%), while the variance explained for family support (45%) and referrals (39%) was considerably higher. This suggests that there may be other characteristics that could have been explored, such as a) types of clinics (inpatient or outpatient), b) types of services (substance abuse, physical health, and mental health), c) characteristics of the illness (acute or long-term), d) workers’ background (e.g., parenting status), and e) characteristics of the families (e.g., age of children), which may be important for the levels of conversations with parents, children, and families. These weaknesses in including possible predictors may be areas for future research.

## Conclusion

There were clear differences in FFP, with generally better scores from social workers and nurses than from psychologists and physicians, which highlights the need for multidisciplinary teams and to strengthen FFP in undergraduate and postgraduate training, especially for psychologists and physicians. CRP scored higher than other clinicians on knowledge and skills, confidence, and referrals, controlling for confounders such as having received specific training. This suggests that highly skilled and motivated champions have been appointed to this change agent role. Establishing this new role may provide important contributions on supervision, especially as co-worker support was the only predictor of having conversations with children. Predictors of FFP varied between the less complex (talking with parents) and the more complex practices (family support and referrals). Both organisation-related factors and worker-related factors were key predictors of family support and referrals, and organisation-related factors explained more of the variance. The findings highlight the need to secure both organisation-related factors and worker-related factors to be able to support families and children. This study confirms many of the predictors of family support and referrals previously found in mental health studies, suggesting that these factors are also valid across different types of services in hospitals.

## Supplementary information

**Additional file 1: **Predictors of Family Focused Practice Behaviours. **Tables S1.** a-e, with both significant and non-significant predictors.

## Data Availability

Data is stored at the Akershus University Hospital. The datasets generated and/or analysed during the current study are not publicly available as it is part of a PhD thesis, and the candidate currently works with the remaining data, but are available from the corresponding author on reasonable request.

## Abbreviations

C: Clinicians
CHIP study: Children of Ill Parents study
CRP: Child Responsible Personnel
EBP: Evidence-Based Practice
FFP: Family Focused Practice
FFPQ: Family Focused Mental Health Practice Questionnaire
H-CRP: Hospital coordinators

## References

[1] Bayer J, Hiscock H, Scalzo K, Mathers M, McDonald M, Morris A (2009). Systematic review of preventive interventions for children's mental health: what would work in Australian contexts?. Aust N Z J Psychiatry.

[2] Hosman CM, van Doesum KT, van Santvoort F (2009). Prevention of emotional problems and psychiatric risks in children of parents with a mental illness in the Netherlands: I. the scientific basis to a comprehensive approach. Aust E-J Adv Ment Health.

[3] Gladstone TR, Beardslee WR, O'Connor EE (2011). The prevention of adolescent depression. Psychiatr Clin North Am.

[4] Hjern A, Berg L, Arat A, Klöfvermark J, Manhica H, Rostila M (2017). Children as next of kin in Sweden.

[5] Leijdesdorff S, van Doesum K, Popma A, Klaassen R, van Amelsvoort T (2017). Prevalence of psychopathology in children of parents with mental illness and/or addiction: an up to date narrative review. Curr Opin Psychiatry.

[6] Pakenham KI, Cox S (2014). The effects of parental illness and other ill family members on the adjustment of children. Ann Behav Med.

[7] Ferm Ulrika, Nilsson Stefan, Nolbris Margaretha Jenholt, Linnsand Petra, Jonsson Annikki (2018). Impact of a Parent’s Neurodegenerative Disease and Care on the Daily Life of Children. Caregiving and Home Care.

[8] Uccelli MM (2014). The impact of multiple sclerosis on family members: a review of the literature. Neurodegener Dis Manag.

[9] Helse- og omsorgsdepartementet (2009). Ot.prp. nr. 84. (2008–2009) Om lov om endringar i helsepersonell loven m.m. (oppfølging av born som pårørande) (About changes in the Health Personnel Act, children as next of kin).

[10] Helsedirektoratet (2010). Barn som pårørende (Children as next of kin).

[11] Skogøy BE, Maybery D, Ruud T, Sørgaard K, Peck GC, Kufås E (2018). Differences in implementation of family focused practice in hospitals: a cross-sectional study. Int J Ment Heal Syst.

[12] Solantaus T, Puras D (2010). Caring for children of parents with mental health problems — a venture into historical and cultural processes in Europe. Int J Ment Health Promot.

[13] Nationellt kompetenscentrum anhoriga (2014). Om vårt uppdrag (About our mission - National competence centrum next of kin) Nka.

[14] Foster K, Maybery D, Reupert A, Gladstone B, Grant A, Ruud T (2016). Family-focused practice in mental health care: an integrative review. Child Youth Serv.

[15] Goodyear M, Hill TL, Allchin B, McCormick F, Hine R, Cuff R (2015). Standards of practice for the adult mental health workforce: meeting the needs of families where a parent has a mental illness. Int J Ment Health Nurs.

[16] Beardslee WR, Gladstone TRG, Wright EJ, Cooper AB (2003). A family-based approach to the prevention of depressive symptoms in children at risk: evidence of parental and child change. Pediatrics.

[17] Beardslee WR, Wright EJ, Gladstone TRG, Forbes P (2007). Long-term effects from a randomized trial of two public health preventive interventions for parental depression. J Fam Psychol.

[18] Beardslee W, Lester P, Klosinski L, Saltzman W, Woodward K, Nash W (2011). Family-centered preventive intervention for military families: implications for implementation science. Prev Sci.

[19] Falkov A, Goodyear M, Hosman CMH, Biebel K, Skogøy BE, Kowalenko N (2016). A systems approach to enhance global efforts to implement family-focused mental health interventions. Child Youth Serv.

[20] Maybery D, Foster K, Goodyear M, Grant A, Tungpunkom P, Skogøy BE, et al. How can we make the psychiatric workforce more family focused? In: Reupert A, Maybery D, Nicholson J, Gopfert M, Seeman M, editors. Parental psychiatric disorder: Distressed parents and their families. 3rd Edition. Cambridge: Cambridge University Press; 2015.

[21] Nicholson J, Reupert A, Grant A, Lees R, Maybery D, Mordoch E, et al. The policy context and change for families living with parental mental illness. In: Reupert A, Maybery D, Nicholson J, Gopfert M, Seeman M, editors. Parental psychiatric disorder: Distressed parents and their families. 3rd Edition. Cambridge: Cambridge University Press; 2015.

[22] Solantaus T, Paavonen EJ, Toikka S, Punamäki R-L. Preventive interventions in families with parental depression: children’s psychosocial symptoms and prosocial behaviour. European child & adolescent psychiatry. 2010;19(12):883-92. 10.1007/s00787-010-0135-3 PMC2988995 20890622

[23] Hjern A, Arat A, Vinnerljung B. Att växa upp med föräldrar som har missbruksproblem eller psykisk sjukdom – hur ser livet ut i ung vuxen ålder? Projektet “Barn som anhöriga”. http://www.anhoriga.se: CHESS, Stockholms universitet/Karolinska Institutet i samarbete med Institutionen för socialt arbete vid Stockholms universitet.; 2014.

[24] Järkestig Berggren U, Hanson E. Stödprogram riktade till barn och/eller föräldrar när en förälder missbrukar alkohol eller andra droger-en kunskapsöversikt. Sweden: Nationellt kompetenscentrum anhöriga; 2016.

[25] Gottfredson D, Kumpfer K, Polizzi-Fox D, Wilson D, Puryear V, Beatty P (2006). The strengthening Washington DC families project: a randomized effectiveness trial of family-based prevention. Prev Sci.

[26] Bröning S, Kumpfer K, Kruse K, Sack P-M, Schaunig-Busch I, Ruths S (2012). Selective prevention programs for children from substance-affected families: a comprehensive systematic review. Subst Abuse Treat Prev Policy.

[27] Järkestig Berggren U, Hanson E (2015). Children as next of kin: a scoping review of support interventions for children who have a parent with a serious physical illness. Child Care Pract.

[28] Niemela M, Hakko H, Rasanen S (2010). A systematic narrative review of the studies on structured child-centred interventions for families with a parent with cancer. Psycho-oncology.

[29] Kühne F, Haagen M, Baldus C, Diareme S, Grether A, Schmitt F (2013). Implementation of preventive mental health services for children of physically ill parents: experiences in seven European countries and health care systems. Gen Hosp Psychiatry.

[30] Reupert A, Maybery D, Cox M, Scott SE (2015). Place of family in recovery models for those with a mental illness. Int J Ment Health Nurs.

[31] MAYBERY D., REUPERT A. (2009). Parental mental illness: a review of barriers and issues for working with families and children. Journal of Psychiatric and Mental Health Nursing.

[32] Maybery D, Reupert A (2006). Workforce capacity to respond to children whose parents have a mental illness. Aust N Z J Psychiatry.

[33] Maybery D, Goodyear M, Reupert A (2012). The family-focused mental health practice questionnaire. Arch Psychiatr Nurs.

[34] Dunst CJ, Trivette CM, Hamby DW (2007). Meta-analysis of family-centered helpgiving practices research. Ment Retard Dev Disabil Res Rev.

[35] Helsedirektoratet (2018). Pårørendeveileder, Veileder om pårørende i Helse- og omsorgstjenester (Recommedations about next of kin in Health- and care services).

[36] Siegenthaler E, Munder T, Egger M (2012). Effect of Preventive Interventions in Mentally Ill Parents on the Mental Health of the Offspring: Systematic Review and Meta-Analysis. J Am Acad Child Adolesc Psychiatry.

[37] Inhestern L, Haller A-C, Wlodarczyk O, Bergelt C (2016). Psychosocial interventions for families with parental cancer and barriers and facilitators to implementation and use–a systematic review. PLoS One.

[38] Rogers EM (2003). Diffusion of innovations.

[39] Rogers EM (2002). Diffusion of preventive innovations. Addict Behav.

[40] Aarons GA, Hurlburt M, Horwitz SM (2011). Advancing a conceptual model of evidence-based practice implementation in public service sectors. Admin Pol Ment Health.

[41] Ploeg J, Davies B, Edwards N, Gifford W, Miller PE (2007). Factors influencing best-practice guideline implementation: lessons learned from administrators, nursing staff, and project leaders. Worldviews Evid-Based Nurs.

[42] Fixsen DL, Naoom SF, Blase KA, Friedman RM, Wallace F (2005). Implementation research: a synthesis of the literature.

[43] Ogden T, Fixsen DL (2015). Implementation science. Z Psychol.

[44] Battilana J, Casciaro T (2012). Change agents, networks, and institutions: a contingency theory of organizational change. Acad Manag J.

[45] Ruud T, Birkeland B, Faugli A, Hagen KA, Hellman A, Hilsen M, et al. Barn som pårørende -Resultater fra en multisenterstudie (Children as next of kin -Results from a multicenter study) IS-05022. Oslo: Akershus universitetssykehus HF; 2015.

[46] Skogøy BE, Sørgaard K, Maybery D, Ruud T, Stavnes K, Kufås E (2018). Hospitals implementing changes in law to protect children of ill parents: a cross-sectional study. BMC Health Serv Res.

[47] Toikka S, Solantaus T (2006). The effective family Programme II: Clinicians' experiences of training in Promotive and preventative child mental health methods. Int J Ment Health Promot.

[48] Region Jönköpings län (2018). Barndialogen.

[49] Maybery D, Goodyear M, O'Hanlon B, Cuff R, Reupert A (2014). Profession differences in family focused practice in the adult mental health system. Fam Process.

[50] Tungpunkom P, Maybery D, Reupert A, Kowalenko N, Foster K (2017). Mental health professionals’ family-focused practice with families with dependent children: a survey study. BMC Health Serv Res.

[51] Whitham J, Eddy K, Maybery D, Reupert A, Fudge E (2009). Use of a web-based delphi study in the development of a training resources for workers supporting families where parents experience mental illness. Int J Ment Health Promot.

[52] Romer G, Saha R, Haagen M, Pott M, Baldus C, Bergelt C (2007). Lessons learned in the implementation of an innovative consultation and liaison service for children of cancer patients in various hospital settings. Psycho-oncology.

[53] Gullbrå F, Smith-Sivertsen T, Rortveit G, Anderssen N, Hafting M (2016). Ill and substance-abusing parents: how can the general practitioner help their children? A qualitative study. BMC Fam Pract.

[54] Aarons GA, Glisson C, Green PD, Hoagwood K, Kelleher KJ, Landsverk JA, et al. The organizational social context of mental health services and clinician attitudes toward evidence-based practice: a United States national study. Implementation science: IS. 2012;7(1):56. 10.1186/1748-5908-7-56 PMC3444886 22726759

[55] Maybery D, Goodyear M, Reupert A, Grant A (2016). Worker, workplace or families: what influences family focused practices in adult mental health?. J Psychiatr Ment Health Nurs.

[56] Grant A, Goodyear M, Maybery D, Reupert A (2016). Differences between Irish and Australian psychiatric Nurses' family-focused practice in adult mental health services. Arch Psychiatr Nurs.

[57] Goodyear M, Maybery D, Reupert A, Allchin R, Fraser C, Fernbacher S (2017). Thinking families: a study of the characteristics of the workforce that delivers family-focussed practice. Int J Ment Health Nurs.

[58] Grant A, Reupert A, Maybery D, Goodyear M (2018). Predictors and enablers of mental health nurses’ family-focused practice. Int J Ment Health Nurs.

[59] Lauritzen C, Reedtz C, Van Doesum K, Martinussen M (2015). Factors that may facilitate or hinder a family-focus in the treatment of parents with a mental illness. J Child Fam Stud.

[60] Houlihan D, Sharek D, Higgins A (2013). Supporting children whose parent has a mental health problem: an assessment of the education, knowledge, confidence and practices of registered psychiatric nurses in Ireland. J Psychiatr Ment Health Nurs.

[61] Korhonen T, Vehviläinen-Julkunen K, Pietilä A-M (2008). Do nurses working in adult psychiatry take into consideration the support network of families affected by parental mental disorder?. J Psychiatr Ment Health Nurs.

[62] Schmitt F, Manninen H, Santalahti P, Savonlahti E, Pyrhonen S, Romer G (2007). Children of parents with Cancer: a collaborative project between a child psychiatry clinic and an adult oncology clinic. Clin Child Psychol Psychiatry.

[63] Lauritzen C, Reedtz C (2016). Child responsible personnel in adult mental health services. Int J Ment Health Syst.

[64] Halsa A, Kufås E, Haugland BSM, Ytterhus B, Dyregrov K (2012). De nye vaktbikkjene: Barneansvarlige i helseforetak (the new watchdogs: child responsible personnel in health Authorietes). Barn som pårørende (children as next of kin).

[65] Östman M, Afzelius M (2011). Children’s representatives in psychiatric services: what is the outcome?. Int J Soc Psychiatry.

[66] Birkeland B, Foster K, Selbekk AS, Høie MM, Ruud T, Weimand B (2018). The quality of life when a partner has substance use problems: a scoping review. Health Qual Life Outcomes.

[67] Birkeland B, Weimand B (2015). En kvalitativ undersøkelse av levekår hos voksne pårørende til personer med rusmiddelproblemer.

[68] Birkeland B, Weimand BM, Ruud T, Høie MM, Vederhus J-K (2017). Perceived quality of life in partners of patients undergoing treatment in somatic health, mental health, or substance use disorder units: a cross-sectional study. Health Qual Life Outcomes.

[69] Kufås E, Faugli A, Weimand B (2015). Barn og ungdom som har foreldre med rusmiddelproblemer: en kvalitativ levekårsstudie:«Når jeg ser han, får jeg sånn stikk i hjertet...».

[70] Hagen KA, Hilsen M, Kallander EK, Ruud T (2019). Health-related quality of life (HRQoL) in children of ill or substance abusing parents: examining factor structure and sub-group differences. Qual Life Res.

[71] Kallander EK, Weimand B, Ruud T, Becker S, Van Roy B, Hanssen-Bauer K. Outcomes for children who care for a parent with a severe illness or substance abuse. Child & Youth Services. 2018;39(4):228-49.

[72] Kallander EK, Weimand BM, Becker S, Van Roy B, Hanssen-Bauer K, Stavnes K (2018). Children with ill parents: extent and nature of caring activities. Scand J Caring Sci.

[73] Statistisk Sentralbyrå (2014). Statistikkbanken.

[74] Laletas S, Goodyear M, Reupert A (1650). Parental mental illness: cross-sectional analysis of family focused practice within the early childhood sector. J Child Fam Stud.

[75] Tchernegovski P, Reupert A, Maybery D (2015). “Let's talk about children”: a pilot evaluation of an e-learning resource for mental health clinicians. Clin Psychol.

[76] Lauritzen C, Reedtz C, Rognmo K, Nilsen MA, Walstad A (2018). Identification and support for children of mentally ill parents: a five year follow-up study of adult mental health services. Front Psychiatry.

[77] Brislin RW (1970). Back-translation for cross-cultural research. J Cross-Cult Psychol.

[78] DeVellis RF. Scale development: Theory and applications. California: Sage publications; 2016.

[79] Nunnally JC (1978). Psychometric theory.

[80] Field A. Discovering statistics using IBM SPSS statistics. California: Sage; 2013.

[81] Cohen A, Doveh E, Eick U (2001). Statistical properties of the rwg(J) index of agreement. Psychol Methods.

[82] Egeland KM, Ruud T, Ogden T, Lindstrøm JC, Heiervang KS. Psychometric properties of the Norwegian version of the Evidence-Based Practice Attitude Scale (EBPAS): to measure implementation readiness. Health research policy and systems. 2016;14(1):47. 10.1186/s12961-016-0114-3 PMC4912744 27316675

[83] Aarons GA (2004). Mental health provider attitudes toward adoption of evidence-based practice: the evidence-based practice attitude scale (EBPAS). Ment Health Serv Res.

[84] Svalheim A-K, Steffenak AKM (2016). Barneansvarliges rolle, knyttet til barn som er pårørende til foreldre med psykiske lidelser – en fenomenografisk studie (Child responsible personnel's role - a phenomenological study). Nordisk Tidsskrift Helseforskning.

[85] Lauritzen Camilla, Reedtz Charlotte (2013). Support for children of service users in Norway. Mental Health Practice.

[86] Ogden T, Bjornebekk G, Kjobli J, Patras J, Christiansen T, Taraldsen K (2012). Measurement of implementation components ten years after a nationwide introduction of empirically supported programs--a pilot study. Implement Sci.

[87] Korhonen T, Pietila AM, Vehvilainen-Julkunen K (2010). Are the children of the clients' visible or invisible for nurses in adult psychiatry?--a questionnaire survey. Scand J Caring Sci.

[88] Korhonen T, Vehvilainen-Julkunen K, Pietila AM (2008). Implementing child-focused family nursing into routine adult psychiatric practice: hindering factors evaluated by nurses. J Clin Nurs.

[89] Frazier PA, Tix AP, Barron KE (2004). Testing moderator and mediator effects in counseling psychology research. J Couns Psychol.

[90] Gjersing L, Caplehorn JR, Clausen T (2010). Cross-cultural adaptation of research instruments: language, setting, time and statistical considerations. BMC Med Res Methodol.

[91] Leonard RA, Linden M, Grant A (2018). Psychometric evaluation of the family focused mental health practice questionnaire in measuring home visitors’ family focused practice. PLoS One.

