# Supplementary material for: Predictors of family focused practice: organisation, profession, or the role as child responsible personnel?
Source: BMC Health Serv Res. 2019 Nov 5;19:793. doi: 10.1186/s12913-019-4553-8 (PMC6829823; doi:10.1186/s12913-019-4553-8)
Supplement: Supplementary file 1 — Additional file 1: Predictors of Family Focused Practice Behaviours. Tables S1. a-e, with both significant and non-significant predictors. [file 12913_2019_4553_MOESM1_ESM.docx]

**Additional file 1**

Predictors of Family Focused Practice Behaviours

Tables S1 a-e, with both significant and non-significant predictors

| **Table S1a**  *Predictors of Conversations with Parents about children’s needs (N =280)* | | | | | | | |
| --- | --- | --- | --- | --- | --- | --- | --- |
|  | *B* | *B SE* | *β* | *Adj. R^2^* | *df* | *F* | *p* |
| (Constant) | 32.839 | 13.614 |  | .211 | 16.216 | 4.861 | .001 |
| Workplace Support | .057 | .043 | .091 |  |  |  | .183 |
| Co-worker Support | -.019 | .062 | -.022 |  |  |  | .753 |
| Time FamilyWork | -.006 | .049 | -.009 |  |  |  | .897 |
| Service Available | -.007 | .050 | -.009 |  |  |  | .892 |
| Spesific Training | .027 | .078 | .023 |  |  |  | .729 |
| Psychologist Dummy | .338 | .176 | .153 |  |  |  | 056 |
| Physician Dummy | .370 | .220 | .126 |  |  |  | .093 |
| Sos. Worker Dummy | .240 | .185 | .087 |  |  |  | .196 |
| Other Dummy | -.067 | .192 | -.023 |  |  |  | .730 |
| Role CRP or C | -.064 | .046 | -.098 |  |  |  | .165 |
| Gender (female) | .415 | .149 | .176 |  |  |  | .006 ** |
| Experience | -.017 | .007 | -.178 |  |  |  | .012 * |
| Knowledge Skills | .308 | .093 | .314 |  |  |  | .001 *** |
| Connectedness | .144 | .081 | .139 |  |  |  | .076 |
| Confidence | .023 | .063 | .027 |  |  |  | .719 |
| Training | .048 | .060 | .053 |  |  |  | .426 |
| *Note*, *R^2^* = .270, *** *p* < .001, ** *p* < 0.1, * *p* < 0.5 | | | | | | | |

| **Table S1b**  *Predictors of Conversations with Children (N =280)* | | | | | | | |
| --- | --- | --- | --- | --- | --- | --- | --- |
|  | *B* | *B SE* | *β* | *Adj. R^2^* | *df* | *F* | *p* |
| (Constant) | 7.176 | 9.950 |  | .109 | 16.216 | 2.762 | .000 |
| Workplace Support | .019 | .031 | .045 |  |  |  | .533 |
| Co-worker support | .094 | .045 | .156 |  |  |  | .039 * |
| Time Family Work | -.003 | .036 | -.007 |  |  |  | .926 |
| Service Available | -.058 | .036 | -.117 |  |  |  | .110 |
| Spesific Training | .048 | .057 | .060 |  |  |  | .399 |
| Psychologist Dummy | -.010 | .129 | -.006 |  |  |  | .941 |
| Physician Dummy | .195 | .160 | .097 |  |  |  | .225 |
| Sos. Worker Dummy | .177 | .135 | .094 |  |  |  | .192 |
| Other Dummy | .009 | .141 | .005 |  |  |  | .948 |
| Role CRP or C | .040 | .033 | .089 |  |  |  | .236 |
| Gender | .068 | .109 | .042 |  |  |  | .534 |
| Experience. | -.004 | .005 | -.064 |  |  |  | .389 |
| Knowledge Skills | .051 | .068 | .076 |  |  |  | .453 |
| Connectedness | .099 | .059 | .139 |  |  |  | .095 |
| Confidence | .069 | .046 | ,117 |  |  |  | .135 |
| Training | .025 | .044 | .040 |  |  |  | .570 |
| *Note,* *R^2^* = .170, *** *p* < .001, ** *p* < 0.1, * *p* < 0.5 | | | | | | | |

| **Table S1c**  *Parents Refusing Conversations with Children (N =280)* | | | | | | | | | | |
| --- | --- | --- | --- | --- | --- | --- | --- | --- | --- | --- |
|  | *B* | *B SE* | *β* | *Adj R^2^* | *df* | *F* | *p* |  |  |  |
| (Constant) | -21.620 | 10.702 |  | .095 | 16.216 | 2.566 | .001 |  |  |  |
| Workplace Support | .003 | .034 | .007 |  |  |  | .924 |  |  |  |
| Co-worker Support | -.083 | .049 | -,129 |  |  |  | .090 |  |  |  |
| Time Family Work | -.056 | .038 | -.113 |  |  |  | .145 |  |  |  |
| Service Available | .041 | .039 | .077 |  |  |  | .294 |  |  |  |
| Spesific Training | .081 | .062 | .095 |  |  |  | .189 |  |  |  |
| Psychologist Dummy | .109 | .138 | .067 |  |  |  | .431 |  |  |  |
| Physician Dummy | .140 | .173 | .065 |  |  |  | .419 |  |  |  |
| Sos. Worker Dummy | -.007 | .145 | -.003 |  |  |  | .964 |  |  |  |
| Other Dummy | -.054 | .151 | -.025 |  |  |  | .722 |  |  |  |
| Role CRP or C | .028 | .036 | .058 |  |  |  | .442 |  |  |  |
| Gender | .278 | .117 | .160 |  |  |  | .018* |  |  |  |
| Experience | .011 | .005 | .147 |  |  |  | .051 |  |  |  |
| Knowledge Skills | .228 | .073 | .316 |  |  |  | .002** |  |  |  |
| Connectedness | -.087 | .063 | -.115 |  |  |  | .169 |  |  |  |
| Confidence | .037 | .050 | .059 |  |  |  | .453 |  |  |  |
| Training | -.008 | .047 | -.012 |  |  |  | .861 |  |  |  |
| *Note,* *R^2^* =.160, *** *p* < .001, ** *p* < 0.1, * *p* < 0.5 | | | |  |  |  |  |  |  |  |

| **Table S1d**  *Predictors of Family support (N =280)* | | | | | | | |
| --- | --- | --- | --- | --- | --- | --- | --- |
|  | *B* | *B SE* | *Β* | *Adj. R^2^* | *df* | *F* | *p* |
| (Constant) | -11.030 | 15.239 |  | .454 | 16.201 | 12.294 | .000 |
| Workplace Support | .024 | .049 | .029 |  |  |  | .625 |
| Co-worker Support | .158 | .068 | .143 |  |  |  | .021* |
| Time Family Work | .183 | .054 | .211 |  |  |  | .001*** |
| Service Available | .079 | .054 | .085 |  |  |  | .148 |
| Spesific Training | .290 | .087 | .193 |  |  |  | .001*** |
| Psychologist Dummy | -.500 | .197 | -.178 |  |  |  | .012* |
| Physician Dummy | .396 | .240 | .107 |  |  |  | .101 |
| Sos. Worker Dummy | .648 | .203 | .187 |  |  |  | .002** |
| Other Dummy | -.124 | .211 | -.033 |  |  |  | .559 |
| Role CRP or C | -.036 | .050 | -.044 |  |  |  | .473 |
| Gender | ,379 | ,163 | ,126 |  |  |  | ,021* |
| Experience | .005 | .008 | .037 |  |  |  | .545 |
| Knowledge Skills | .171 | .102 | .137 |  |  |  | .096 |
| Connectedness | .181 | .089 | .136 |  |  |  | .045* |
| Confidence | .068 | .070 | .062 |  |  |  | .333 |
| Training | .082 | .068 | .069 |  |  |  | .226 |
| *Note,* *R^2^* ^=^  .495, *** *p* < .001, ** *p* < 0.1, * *p* < 0.5 | | | | | | | |

| **Table S1e**  *Predictors of Referrals (N = 280)* | | | | | | | |
| --- | --- | --- | --- | --- | --- | --- | --- |
|  | *B* | *B SE* | *β* | *Adj. R^2^* | *df* | *F* | *p* |
| (Constant) | 9.526 | 20.596 |  | .387 | 16.186 | 9.122 | .000 |
| Workplace Support | .076 | .067 | .075 |  |  |  | .257 |
| Co-worker Support | .054 | .093 | .039 |  |  |  | .563 |
| Time Family Work | .114 | .074 | .108 |  |  |  | .125 |
| Service Available | .326 | .074 | .288 |  |  |  | .000*** |
| Spesific Training | .003 | .119 | .002 |  |  |  | .979 |
| Psychologist Dummy | -.404 | .263 | -.116 |  |  |  | .127 |
| Physician Dummy | -.033 | .327 | -.007 |  |  |  | .920 |
| Sos. Worker Dummy | .601 | .281 | .138 |  |  |  | .034* |
| Other Dummy | -.050 | .277 | -.011 |  |  |  | .857 |
| Role CRP or C | .075 | .067 | .073 |  |  |  | .261 |
| Gender | .333 | .219 | .091 |  |  |  | .130 |
| Experience | -.006 | .010 | -.037 |  |  |  | .578 |
| Knowledge Skills | .293 | .142 | .189 |  |  |  | .041* |
| Connectedness | .033 | .117 | .021 |  |  |  | .781 |
| Confidence | .103 | .096 | .075 |  |  |  | .284 |
| Training | .094 | .088 | .066 |  |  |  | .289 |
| *Note,* *R^2^* = .440, *** *p* < .001, ** *p* < 0.1, * *p* < 0.5 | | | | | | | |
